# A new species of *Pseudopaludicola* (Anura, Leiuperinae) from Espírito Santo, Brazil

**DOI:** 10.7717/peerj.4766

**Published:** 2018-05-16

**Authors:** Dario E. Cardozo, Diego Baldo, Nadya Pupin, João Luiz Gasparini, Célio F. Baptista Haddad

**Affiliations:** 1 Laboratorio de Genética Evolutiva, Instituto de Biología Subtropical, CONICET-UNaM, Posadas, Misiones, Argentina; 2 Departamento de Zoologia and Centro de Aquicultura (CAUNESP), Universidade Estadual Paulista, Rio Claro, São Paulo, Brazil; 3 Laboratório de Vertebrados Terrestres, Universidade Federal do Espírito Santo, São Mateus, Espírito Santo, Brazil

**Keywords:** Leptodactylidae, Morphology, Taxonomy, 16S rDNA, Advertisement call, Chromosome number

## Abstract

We describe a new anuran species of the genus *Pseudopaludicola* that inhabits sandy areas in resting as associated to the Atlantic Forest biome in the state of Espírito Santo, Brazil. The new species is characterized by: SVL 11.7–14.6 mm in males, 14.0–16.7 mm in females; body slender; fingertips knobbed, with a central groove; hindlimbs short; abdominal fold complete; arytenoid cartilages wide; prepollex with base and two segments; prehallux with base and one segment; frontoparietal fontanelle partially exposed; advertisement call with one note composed of two isolated pulses per call; call dominant frequency ranging 4,380–4,884 Hz; diploid chromosome number 22; and Ag-NORs on 8q subterminal. In addition, its 16S rDNA sequence shows high genetic distances when compared to sequences of related species, which provides strong evidence that the new species is an independent lineage.

## Introduction

The monophyletic genus *Pseudopaludicola* is currently composed of 21 recognized species broadly distributed throughout Colombia, Venezuela, Guiana, southwestern Surinam, northeastern Peru, eastern Bolivia, Paraguay, much of Brazil and northern eastern and central Argentina and Uruguay (Frost, 2018). Lynch (1989) performed a taxonomic revision of the genus and proposed the inclusion of *P. boliviana*, *P. ceratophyes*, *P. llanera*, and *P. pusilla* in a group named the *P. pusilla* group, based on the presence of a T-shaped terminal phalange on Toe IV. Subsequently, Lobo (1995) provided a phylogenetic analysis and a key for the genus, recovering *Pseudopaludicola* supported by the synapomorphies: (1) presence of one antebrachial tubercle; (2) antero- and posterolateral processes of the hyoid vestigial or absent; and (3) narrow epicoracoid cartilages, slightly overlapping each other or not overlapping. The *P. pusilla* group was also recovered as monophyletic, including the same four species and supported by the same synapomorphy previously mentioned by Lynch (1989), with the rest of the species unassigned to any group. Several new species have since been described increasing considerably the known taxonomic diversity of the genus. However, the taxonomy of the genus became unstable and several changes were made: *P. mirandae* was considered as a junior synonym of *P. boliviana* (Cardozo & Lobo, 2009); the type locality and the type series of *P. ternetzi* were updated (Caramaschi & Pombal Jr, 2011; Cardozo & Baldo, 2012); *P. canga*, assigned to the *P. pusilla* group in the original description (Giaretta & Kokubum, 2003), was removed from this group by Cardozo & Suárez (2012); *P. riopiedadensis* was proposed as a junior synonym of *P. ternetzi* (Cardozo & Toledo, 2013); *P. ameghini* was resurrected (Pansonato et al., 2013); the ephemeral *P. serrana* was synonymized with *P. murundu* (Pansonato et al., 2014b); and the validity of *P. parnaiba* was questioned by Carvalho et al. (2015). Additionally, several populations with different karyotypes were reported, suggesting the presence of several innominate taxa (Duarte et al., 2010; Fávero et al., 2011). Meanwhile, a molecular phylogenetic hypothesis was proposed by Veiga-Menoncello et al. (2014), recovering *Pseudopaludicola* as a monophyletic group and supporting the *P. pusilla* group of Lynch (1989) and Lobo (1995), although only two of its five species were included in their analysis. These authors also proposed the *P. saltica* group to include *P. saltica*, *P. murundu* (including *P. serrana*), and *P. jaredi* (as *P.* aff. *saltica*). In addition, a progressive chromosome number reduction was proposed by Veiga-Menoncello et al. (2014), and later Cardozo et al. (2016) pointed out the independent origins of specimens with 2*n* = 20 chromosomes assigned to *P. boliviana* from Argentina and Paraguay.

In the present work, we describe a new species of *Pseudopaluciola* from Espírito Santo State, Brazil, which is clearly distinguishable from other species by a combination of morphological, acoustic and chromosomal characters and supported by molecular evidence from a partial sequence of mitochondrial 16S rDNA.

## Materials & Methods

### Ribosomal DNA (rDNA) sequences

We obtained a partial sequence of the mitochondrial gene 16S (nearly 500 base pairs) from eight specimens from two localities in Espírito Santo Sate, Brazil. Total genomic DNA was extracted from ethanol-preserved tissues (muscle) using the Qiagen DNeasy kit. PCR amplifications were carried out in 30 µl reactions using 0.2 µl Taq (Genbiotech). The mitochondrial gene 16S was amplified using the primer pair 16SAr/16SBr (Palumbi et al., 1991). This fragment was amplified following a PCR protocol consisting of an initial denaturation step at 95 °C (10 min); 35 cycles consisting of 95 °C (30 s) for denaturation, 55 °C (1 min) for annealing, and 72 °C (2 min) for extension; and a final extension step at 72 °C (10 min). PCR-amplified products were purified with an Accuprep purification Kit (Bioneer). The products were sequenced with an automatic sequencer ABI 3730XL (Macrogen, South Korea) and all samples were sequenced in both directions to check for potential errors. Chromatograms obtained from the automated sequencer were processed using Sequencher 4.5 (Gene Codes, Ann Arbor, MI, USA). Complete sequences were edited with BioEdit (Hall, 1999) and deposited in GenBank under the accession numbers (MG825764, MG825766, MG825767).

To estimate genetic distances between *Pseudopaludicola* sp. nov. and related species, sequences of a fragment of the mitochondrial 16S rDNA gene were taken from GenBank (Appendix I) and aligned in MAFFT (Katoh & Toh, 2008) under G-INS-i strategy. We followed the hypothesis of Veiga-Menoncello et al. (2014) to test genetic distances between *P.* sp. nov. versus all *Pseudopaludicola* species with data available. The aligned dataset comprised 575 base pairs and uncorrected pairwise distances were calculated using PAUP* (Swofford, 2002).

### Morphology

Types and referred specimens were deposited in the Célio F.B. Haddad Collection (CFBH), Universidade Estadual Paulista, Campus Rio Claro, São Paulo, Brazil, and Laboratorio de Genética Evolutiva, Instituto de Biología Subtropical, Posadas, Misiones, Argentina (LGE). Additional examined specimens came from the following collections: Academy of Natural Sciences, Philadelphia, USA (ANSP); American Museum of Natural History, New York, USA (AMNH); Centro Nacional de Investigaciones Iológicas, Buenos Aires, Argentina (CENAI, actually housed in MACN); Museu de Zoologia da Universidade Estadual de Campinas “Adão José Cardoso”, Campinas, São Paulo, Brazil (ZUEC); Departamento de Zoologia, Instituto de Biociências, UNESP, Rio Claro, São Paulo, Brazil (CFBH); Fundación Miguel Lillo, San Miguel de Tucumán, Argentina (FML); Instituto de Ciencias Naturales, Universidad Nacional de Bogotá, Colombia (ICN); Instituto Nacional de Pesquisas da Amazônia, Manaus, Amazonas, Brazil (INPA); Museo Argentino de Ciencias Naturales “Bernandino Rivadavia”, Capital Federal, Argentina (MACN); Museu Nacional, Rio de Janeiro, Rio de Janeiro, Brazil (MNRJ); Museu Paraense Emilio Goeldi, Belém, Pará, Brazil (MPEG); Museu de Zoologia, Universidade de São Paulo, São Paulo, Brazil (MZUSP); and Instituto de Investigación Biológica del Paraguay, Asunción, Paraguay (IIBP). See list of specimens in Supplemental Information.

Specimens collected for the present study were euthanized with lidocaine. Some specimens were then fixed in 10% formalin and stored in 70% ethyl alcohol while others were not fixed in formalin and directly stored in 100% ethyl alcohol for DNA analysis. Thirteen morphometric variables were measured: snout-vent length (SVL), head length (HL), head width (HW), eye diameter (ED), interorbital distance (IOD), internarial distance (IND), and tibia length (TL) following Duellman (1970); thigh length (THL) was measured following Heyer et al. (1990), while eye-nostril distance (END), nostril–snout distance (NSD), and foot length (FL) were measured according to Napoli (2005). Two other measurements, length of Finger II (LII), and length of Finger III (LIII), were taken from the base of the proximal subarticular tubercle to the tip of the respective finger. All measurements are given in millimeters (mm) and were recorded with an ocular micrometer in a Nikon SMZ 800 stereomicroscope, except for SVL, which was taken with calipers to the nearest 0.1 mm. In addition, we calculated the cephalic index (CI), defined as head length/head width (HL/HW). Sex was determined by visual inspection of male secondary sexual characters (nuptial pads and/or extended vocal sacs) and the presence of ovarian follicles in females.

The osteological description is based on one adult male (paratype CFBH 37716), previously cleared and stained (CS) using the technique of Taylor & Van Dyke (1985). Osteological terminology follows Trueb (1973) for general features, Jurgens (1971) for the cranium, Trewavas (1933) for the hyoid and larynx, Alberch & Gale (1985) for the phalangeal formula, and Fabrezi (1992); Fabrezi (1993) and Fabrezi & Alberch (1996) for the carpus and tarsus. For comparison, we cleared and stained adult males of *P. ameghini*, *P. boliviana*, *P. canga*, *P. ceratophyes*, *P. falcipes*, *P. giarettai*, *P. llanera*, *P. mineira*, *P. murundu*, *P. mystacalis*, *P. motorzinho*, *P. parnaiba*, *P. pocoto*, *P. pusilla*, *P. saltica*, and *P. ternetzi* (Supplemental Information).

### Advertisement call

We analyzed 250 advertisement calls obtained from three males (LGE 20329, and two unvouchered specimens), near Fazenda Jacuhy, Serra (20°13′41.4″S, 40°20′3.6″W, datum WGS 84; 2 m above sea level [m asl]), Espírito Santo State, Brazil, (19:30–20:15 h, with air temperature [AT] = 27 °C, water temperature [WT] = 28 °C); and one specimen (LGE 20340) recorded at Restinga de Praia das Neves, Presidente Kennedy (21°14′41.53″S, 40°58′45.80″W; 6 m asl) at 20:00 h under AT = 25 °C and WT = 27 °C. Advertisement calls were recorded with a Marantz PMD 660 digital recorder, and an Audiotechnia AT directional microphone. Recordings were analyzed employing Sound Forge pro 11.0 software with a FFT of 512 points, at a sampling rate of 44.1 kHz and 16-bit precision. The following parameters were measured from the waveform: call duration (in milliseconds), interval between calls, number of pulses per call, duration of each pulse, and interpulse interval. Mean power spectra were obtained with a FFT of 512 points, 93% overlap, Hamming’s sampling window and spectrogram resolution of 10,000 samplings. Dominant frequencies were obtained from spectrograms. The call rate (calls per second) was calculated. Terminology for advertisement call descriptions follows Heyer et al. (1990). The advertisement call records are deposited in the sound collection of the Laboratorio de Genética Evolutiva (LGE).

### Cytogenetic analysis

Chromosome spreads were prepared from intestinal epithelium and testes (Schmid et al., 2010). Mitotic chromosome preparations were obtained from four males (LGE 20330, 20332, 20334, and 20336) and three females (LGE 20331, 20333, and 20335). Cellular spreads were stained with a Giemsa-PBS solution (pH 6.8). The silver staining of nucleolar organizer regions (Ag-NORs) and C-banding techniques were performed according to Howell & Black (1980) and Sumner (1972), respectively. The relative length (RL), centromeric index (CI), and centromeric ratio (CR) were scored using the software Micromeasure v3.3 (Reeves & Tear, 2000). Karyotypes were arranged according to decreasing chromosome size, following the nomenclature of Green & Sessions (1991); Green & Sessions (2007), and preserving the apparent homology with data available from the literature. We used x (basic chromosome number), 2n (somatic chromosome number), and FN (fundamental number of chromosome arms) as suggested by White (1954). Other abbreviations used are: NORs (nucleolar organizer regions), p (short arm), q (long arm), and sc (secondary constrictions).

The electronic version of this article in Portable Document Format (PDF) will represent a published work according to the International Commission on Zoological Nomenclature (ICZN), and hence the new name contained in the electronic version is effectively published under that Code from the electronic edition alone. This published work and the nomenclatural act it contains has been registered in ZooBank, the online registration system for the ICZN. The ZooBank LSIDs (Life Science Identifiers) can be resolved and the associated information viewed through any standard web browser by appending the LSID to the prefix “http://zoobank.org/.” The LSID for this publication is: urn:lsid:zoobank.org:pub:114E0628-F00E-42B3-A98E-CB157DCA1CF1. The online version of this work is archived and available from the following digital repositories: PeerJ, PubMed Central and CLOCKSS.

## Results

### Molecular distance matrix

The new species showed a relatively high genetic divergence in relation to other species of *Pseudopaludicola*, but having relatively lower pairwise distances with species having 22-chromosomes (from 3.7 % with *P. pocoto* to ≈7% with *P. falcipes*). The new taxon has genetic distances of greater than 10 % with the remaining species of *Pseudopaludicola* (Table 1).

**Table 1:** Percentage of uncorrected pairwise distance (*p*-distance) between *Pseudopaludicola restinga* sp. nov. and the remaining *Pseudopaludicola* species with data available. GenBank accession number: *P. ameghini* (KJ146975; KJ147047), *P. atragula* (KJ146996), *P. boliviana* (KJ147049– KJ147050), *P. canga* (KJ146988–KJ146990); *P. facureae* (KJ146968); *P. falcipes* (AY843741; KJ146972–KJ146973), *P. jaredi* (KJ147033–KJ147034), *P. llanera* (KP149332; KP149338; KP149453; KP149482; KP149292), *P. mineira* (KJ147025–KJ147027), *P. motorzinho* (KJ146992; KJ147039–KJ147041), *P. murundu* (KJ147008, KJ147030–KJ147031, KJ147051, KJ147053), *P. mystacalis* (KJ146981–KJ146985; KJ146991; KJ146999; KJ147005; KJ147009; KJ147020; KJ147022–KJ147024; KJ147028; KJ147037–KJ147038; KJ147044), *P. pocoto* (KJ1470235–KJ1470236), *P. restinga* sp. nov. (MG825764, MG825766–MG825767), *P. saltica* (KJ146993–KJ146995, KJ140022–KJ140124), *P. ternetzi* (KJ146986–KJ146987; KJ147010; KJ147042–KJ147043; KJ147054–KJ147056).

**Species**	**1**	**2**	**3**	**4**	**5**	**6**	**7**	**8**	**9**	**10**	**11**	**12**	**13**	**14**	**15**	**16**
***P. restinga*sp. nov.**	0.0–0.4															
*P. pocoto*	3.7–4.1	0.0														
*P. mineira*	4.3–4.7	3.8	0.0													
*P. murundu*	5.3–5.8	5.7–5.9	4.5–4.7	0.0–0.4												
*P. jaredi*	6.2–6.9	6.5–7.1	5.4–5.9	2.1–2.5	0.0											
*P. saltica*	6.2–6.9	6.2–6.6	5.0–5.2	2.3–2.5	2.7–2.9	0.0–0.4										
*P. falcipes*	7.4–8.0	7.6–7.8	6.8–7.0	6.7–7.1	8.3–9.1	8.5–8.9	0.0–0.5									
*P. boliviana*	12.4–13.3	12.0–12.6	11.5–12.0	11.4–12.2	12.0–13.5	12.5–13.4	11.4–12.1	0.0–0.5								
*P. motorzinho*	12.9–13.6	13.2–13.4	12.9–13.1	12.0–12.6	12.1–13.3	13.5–13.8	12.9–13.1	7.2–7.6	0.0–0.4							
*P. llanera*	13.6–14.2	13.6–13.8	12.7–13.1	12.1–12.8	13.1–14.7	13.7–14.0	12.9–13.3	6.9–7.3	7.4–8.1	0.0–1.9						
*P. ternetzi*	13.5–14.3	13.8–14.0	12.3–12.6	11.0–11.7	11.7–12.9	12.3–13.0	12.4–13.6	11.7–12.6	11.5–11.7	11.7–12.4	0.0–1.1					
*P. ameghini*	13.5–14.0	13.1–13.3	11.7–11.9	10.9–11.0	11.2–12.2	11.8–12.1	12.4–12.9	12.5–12.8	11.7–11.9	11.7–12.8	1.2–2.1	0.0–0.5				
*P. mystacalis*	15.4–16.3	15.3–15.8	13.5–14.4	12.8–13.9	13.7–15.8	13.9–15.3	13.9–14.9	14.0–15.6	14.3–15.9	14.4–15.8	8.5–10.7	8.4–10.1	0.0–3.2			
*P. canga*	15.7–16.1	16.3	15.1	13.1–13.3	14.2–15.2	14.6–14.8	13.7–14.2	15.6–15.8	15.2–15.5	15.7–16.4	8.4–9.1	8.9–9.1	6.5–8.5	0.0		
*P. atragula*	17.2–17.5	17.0–17.1	15.9	14.1–14.2	14.4–15.7	14.1–14.3	15.6–15.8	17.4–17.6	16.6–17.0	16.3–16.6	10.6–11.0	10.3–10.5	8.3–9.4	7.8	0.0	
*P. facureae*	17.2–17.5	17.0–17.1	16.2	14.2–14.6	14.6–15.9	14.4–14.8	14.9–15.3	16.7	15.7–16.1	15.6–15.9	10.8–11.2	10.5–10.6	9.0–9.6	8.1	3.2	0.0

#### Species Description

**Table:** 

*Pseudopaludicola restinga* sp. nov.
urn:lsid:zoobank.org:act:776C919B-CD34-49AD-8413-FC480407B859
(Figs. 1–9; Table 2)

**Synonyms.** *Pseudopaludicola* aff*. falcipes* Almeida, Gasparini & Peloso (2011): 548 (listed) and Gasparini (2012): 15 (listed).

**Figure 1:**
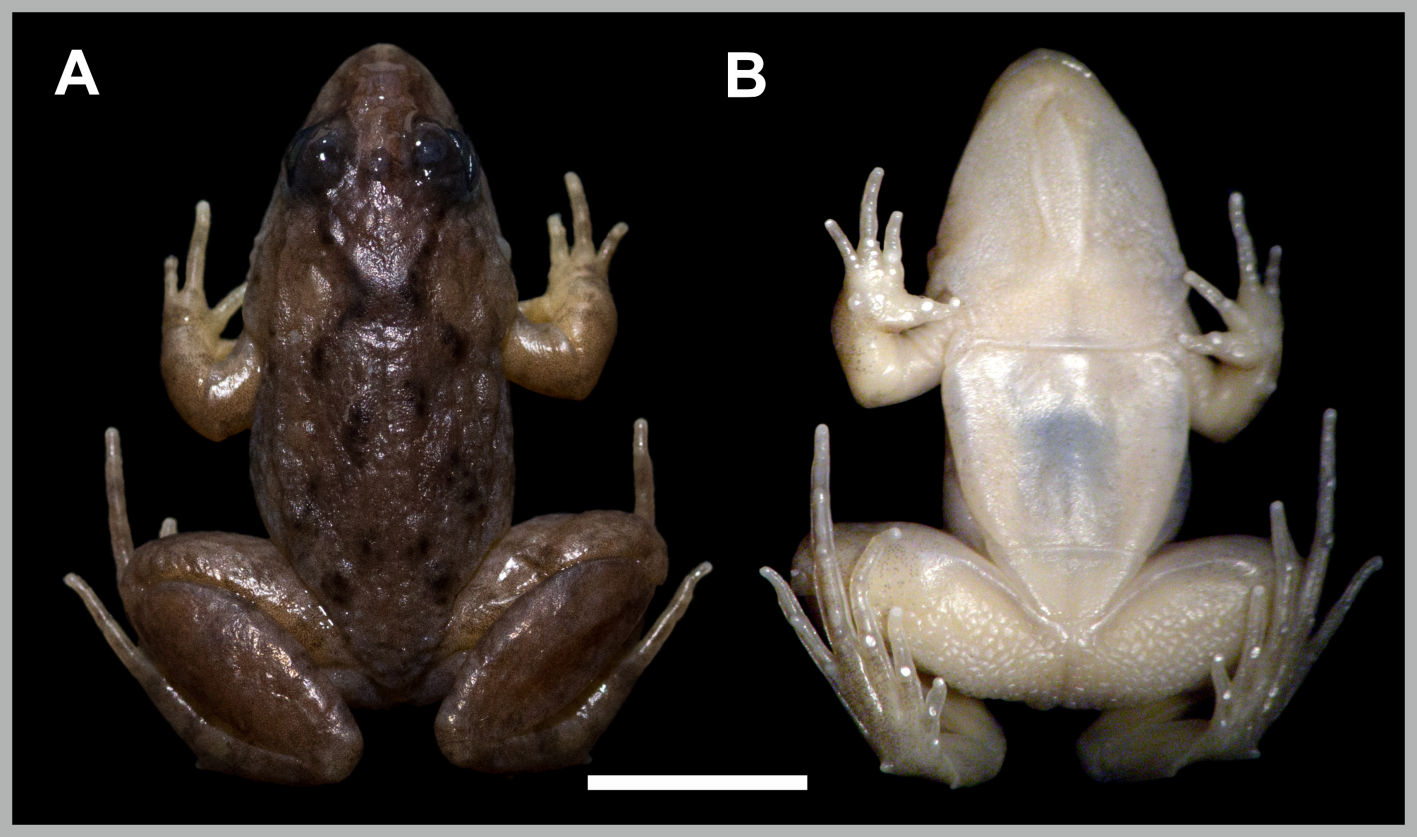
*Pseudopaludicola restinga* sp. nov., holotype, CFBH 37715. (A) Dorsal and (B) ventral views. Scale bar = 5 mm. Photo: D Cardozo.

**Figure 2:**
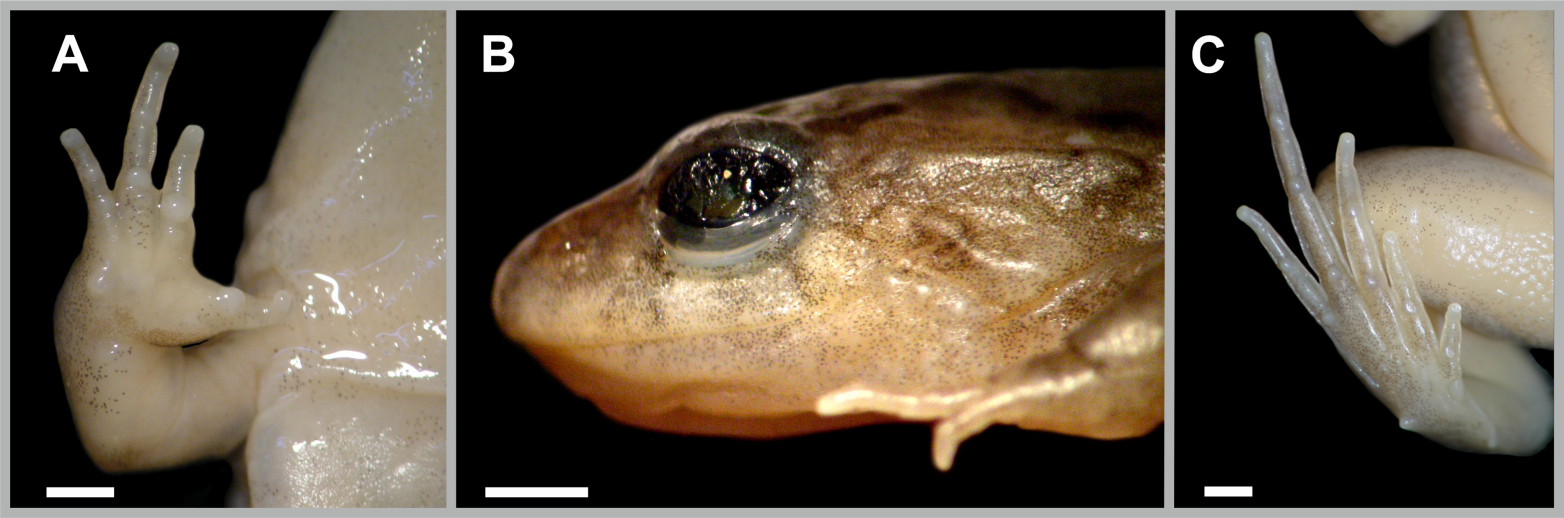
*Pseudopaludicola restinga* sp. nov., holotype, CFBH 37715. Ventral view of right hand (A), lateral view of the head (B), and ventral view of the right foot (B). Scale bar = 1 mm. Photo: D Cardozo.

**Holotype.** CFBH 37715, an adult male collected on February 17, 2009 near Fazenda Jacuhy, municipality of Serra (20°13′41.4″S, 40°20′3.6″W, datum WGS 84; 2 m above sea level [asl]), Espírito Santo State, Brazil, by J.L. Gasparini and R.R. Zorzal (Figs. 1– 2).

**Figure 3:**
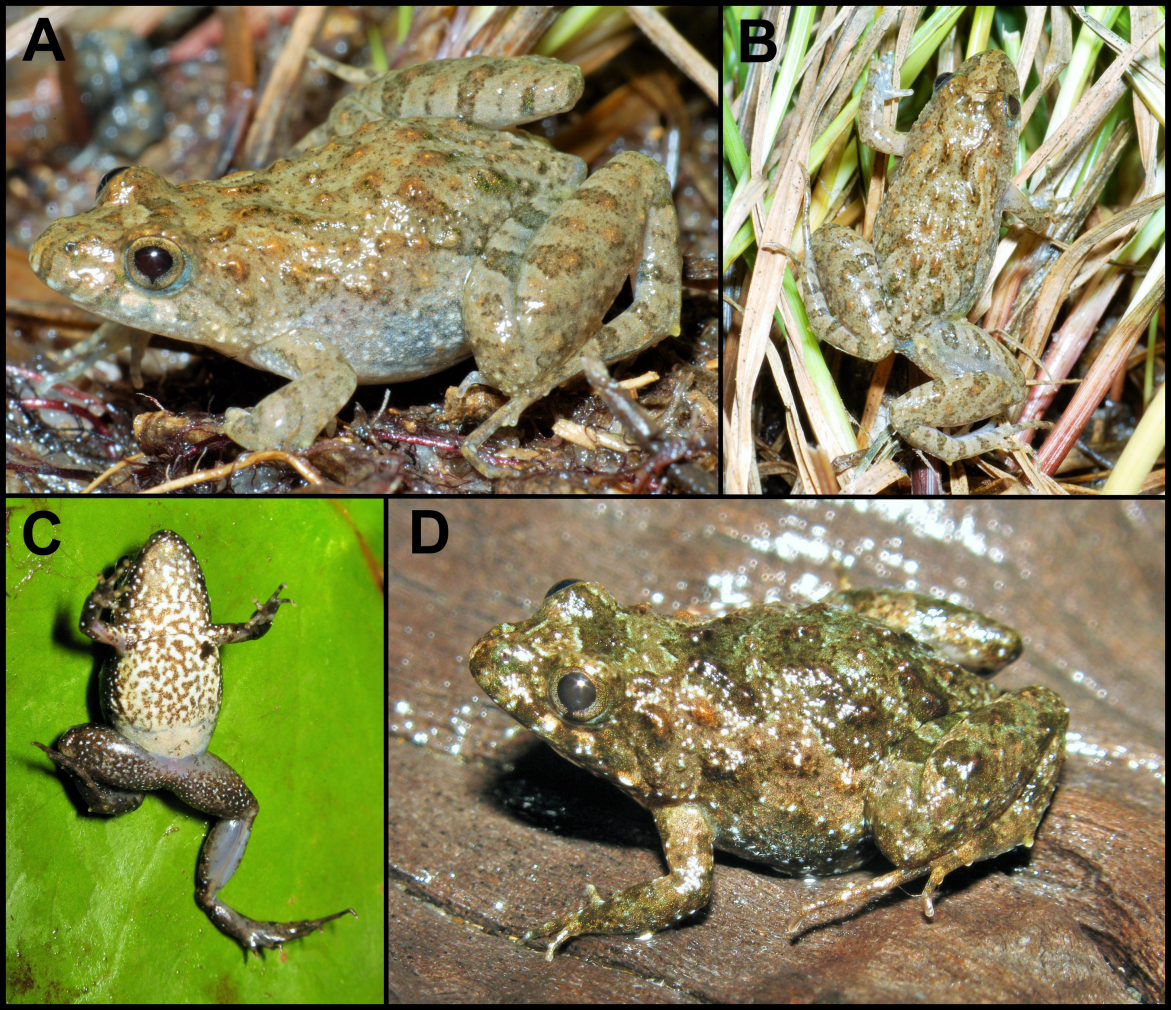
*Pseudopaludicola. restinga* sp. nov. in life. Specimens from Fazenda Jacuhy, Serra (A–B), and Restinga de Praia das Neves, Presidente Kennedy (C–D). Photo: J Gasparini.

**Figure 4:**
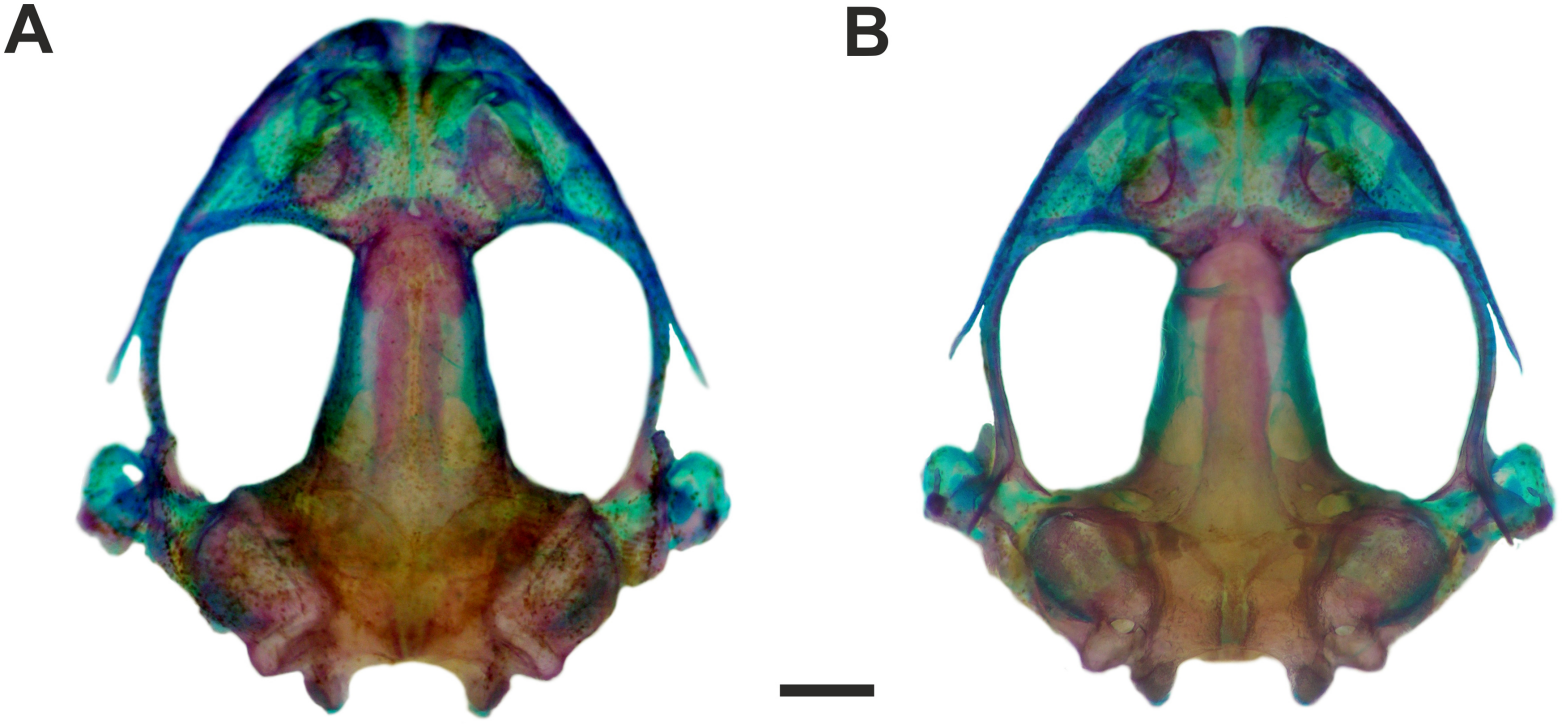
Skull of *Pseudopaludicola. restinga* sp. nov Dorsal (A) and ventral (B) views of the skull of *Pseudopaludicola restinga* sp. nov., paratype CFBH 37716, adult male. Scale bar = 1 mm. Photo: D Cardozo.

**Figure 5:**
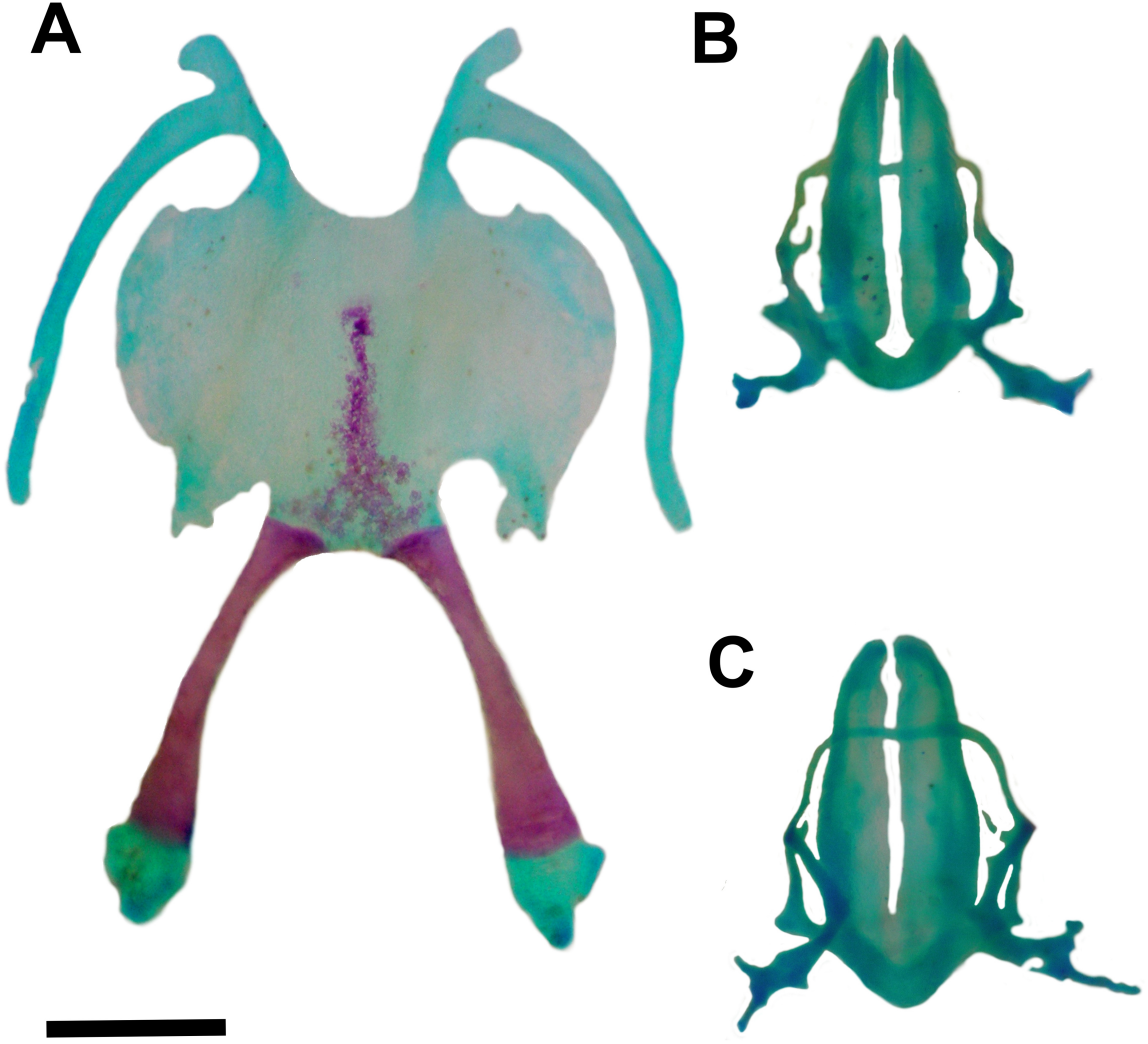
Hyoid and larynx of *Pseudopaludicola. restinga* sp. nov. Hyoid (A), cardiac view of the larynx (B), esophageal view of the larynx (C) of *Pseudopaludicola restinga* sp. nov., paratype, CFBH 37716, adult male. Scale bar = 0.5 mm. Photo: D Cardozo.

**Figure 6:**
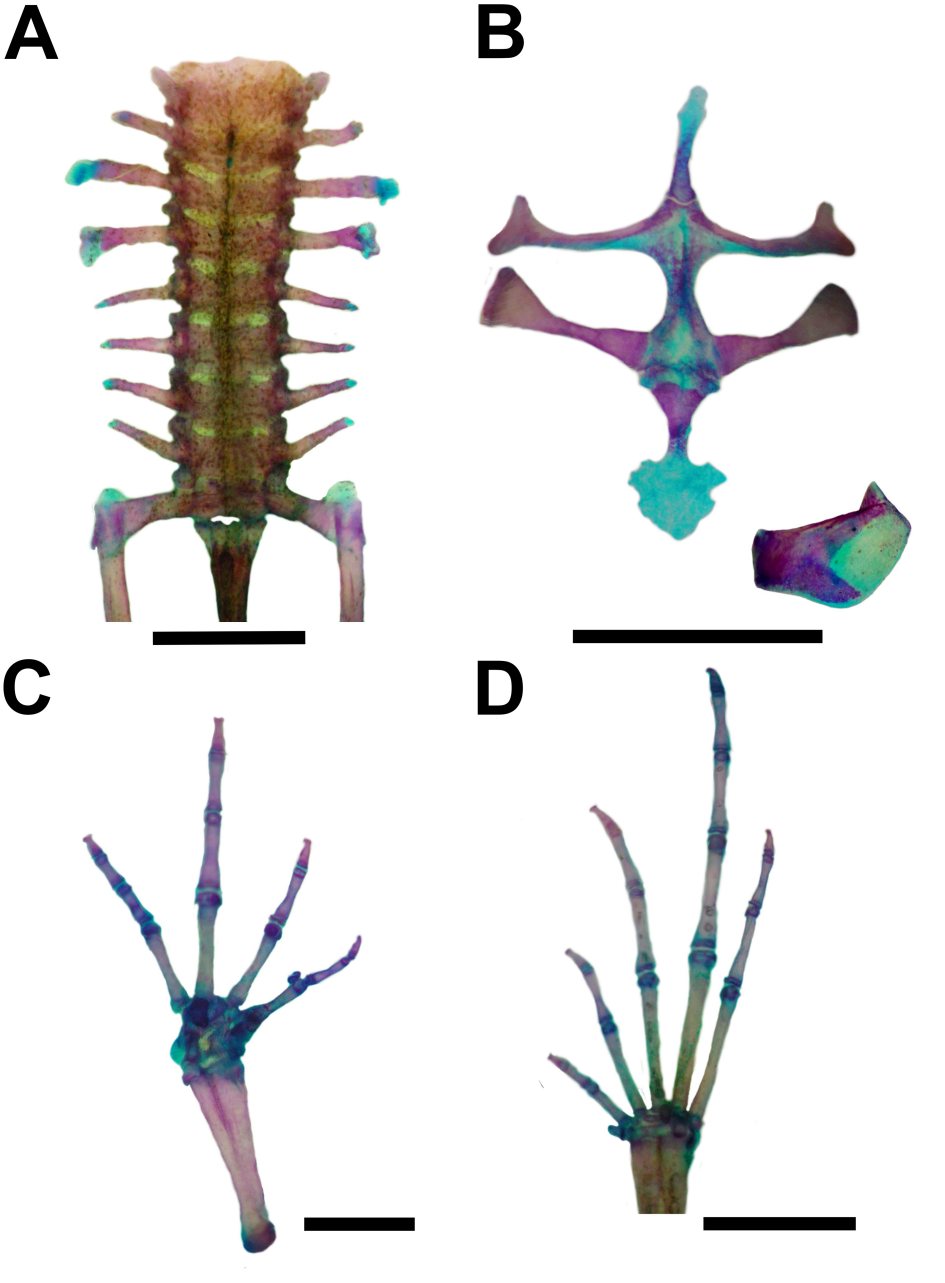
Skeleton of *Pseudopaludicola. restinga* sp. nov., paratype CFBH 37716, adult male. (A) Vertebral column in dorsal view; (B) epicoracoid cartilages, and scapula; (C) palmar view of hand; (D) plantar view of foot. Scale bar = 1 mm. Photo: D Cardozo.

**Figure 7:**
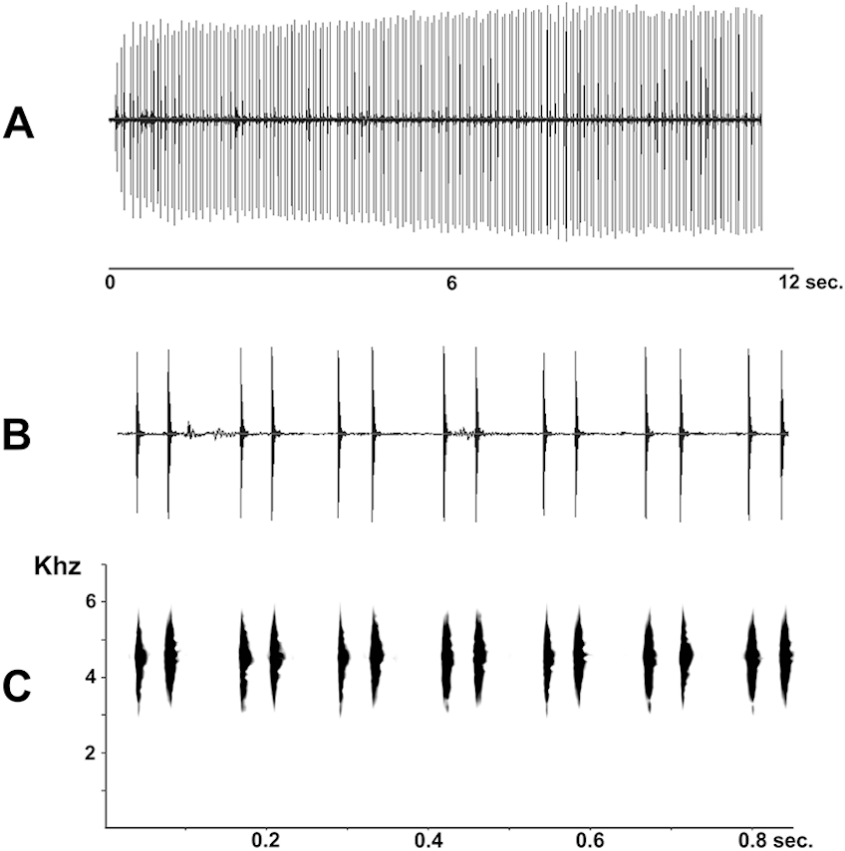
Advertisement call of *Pseudopaludicola restinga* sp. nov. (LGE 20329), oscilogram of a sequence of advertisement calls (A); sonogram detailing seven calls (B); audio spectrogram (C).

**Figure 8:**
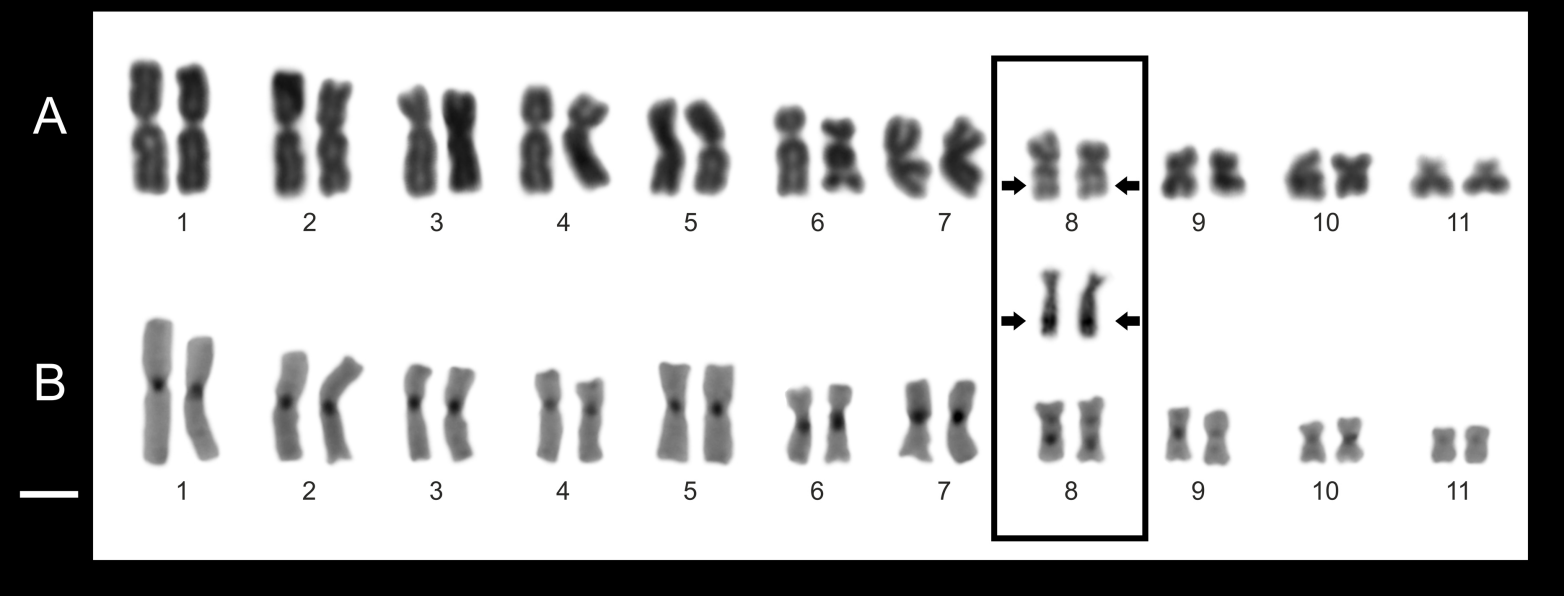
Chromosome features of *Pseudopaludicola.restinga* sp. nov. (LGE 20332). Conventional staining (A); C-banding technique (B). Inset (arrows) NORs bearing pair chromosome. Scale bar = 10 µm.

**Figure 9:**
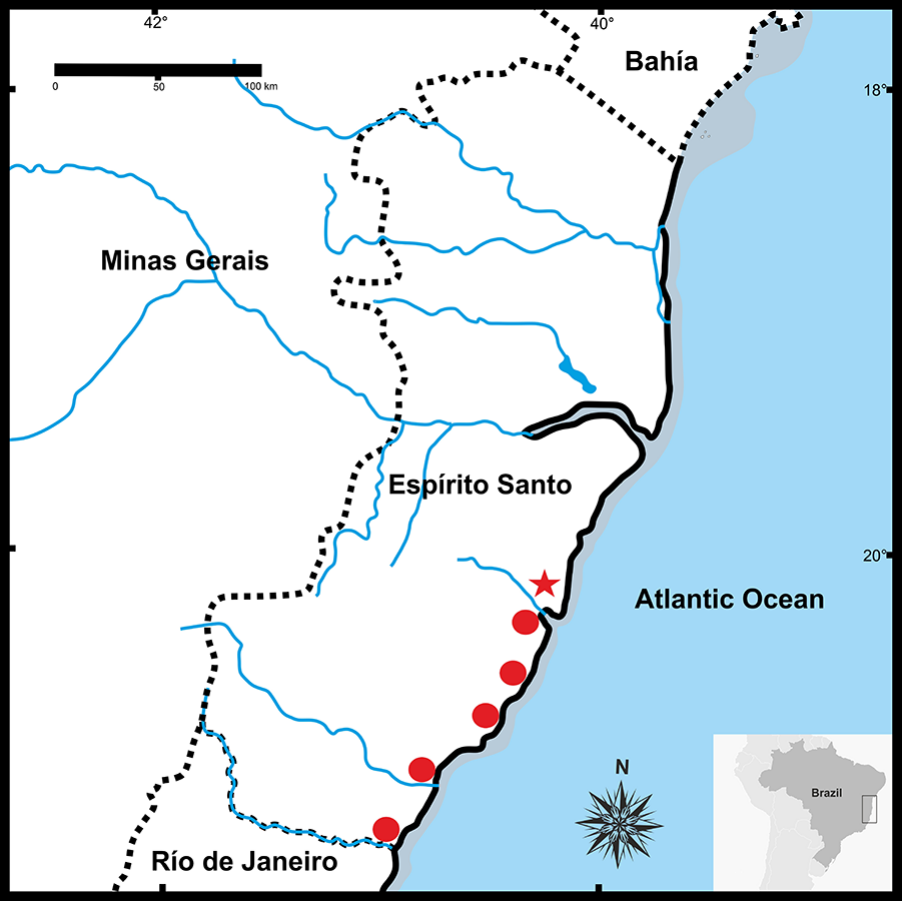
Geographic distribution of *Pseudopaludicolarestinga* sp. nov. The star indicates the type locality.

**Table 2:** Descriptive statistics of adult males and females of Pseudopaludicola restinga sp. nov.

	**Paratypes**							
	Males (*n* = 21)				Females (*n* = 14)			
	Mean	Min	Max	SD	Mean	Min	Max	SD
SVL	13.5	11.7	14.6	0.7	15.4	14.0	16.7	1.0
HL	4.8	4.6	5.0	0.1	5.1	4.5	5.5	0.3
HW	4.3	4.1	4.5	0.1	4.5	4.1	5.0	0.3
ED	1.6	1.2	1.7	0.1	1.7	1.6	1.8	0.1
IOD	1.4	1.2	1.8	0.2	1.4	1.3	1.5	0.1
IND	1.2	1.1	1.4	0.1	1.2	1.1	1.3	0.1
TL	6.8	6.6	7.2	0.2	7.4	6.8	8.1	0.4
THL	6.4	5.8	6.7	0.3	7.0	6.6	8.0	0.4
END	1.0	0.9	1.1	0.1	1.2	1.0	1.3	0.1
NSD	0.7	0.5	0.8	0.1	0.7	0.6	0.8	0.1
FL	7.3	6.6	7.9	0.5	7.9	6.9	8.5	0.4
LII	1.0	0.9	1.2	0.1	1.1	1.0	1.2	0.1
LIII	1.3	1.1	1.4	0.1	1.3	1.3	1.4	0.1

**Notes.**

*n* number of specimens Min minimum Max maximum SD standard deviation

**Paratypes.** CFBH 10817, 10821, 10823, 10825, 10827–8, 10832–3, 37679, 37718 (females); CFBH 10818, 10820, 10826, 10830–1, 10834–5, 37714–7, 37719 (males); CFBH 10819, 10822, 10824, 10829, 10836–7 (juveniles); and CFBH-T 2222–3; 6676–9 (adults preserved in 100% ethanol), with same data as holotype; LGE 20329 (male) collected by D. Cardozo and J.L. Gasparini on December 11 2017; 20330, 20332, 20334, 20336 (males), 20331, 20333, 20335 (females), 20337–8 (two adults males preserved in 100% ethanol) collected by D. Cardozo, J.L. Gasparini, and E. Gasparini on December 13, 2017. CFBH 39228–32 (males) collected in Restinga de Praia das Neves, municipality of Presidente Kennedy (21°14′41.53″S, 40°58′45.80″W; 6 m asl), Espírito Santo State, Brazil, by J.L. Gasparini and CFB Haddad; LGE 20340*–*1 (two adult males preserved in 100% ethanol) collected at Restinga de Praia das Neves, municipality of Presidente Kennedy (21°14′41.53″S, 40°58′45.80″W; 6 m asl), Espírito Santo State, Brazil, by D. Cardozo, J.L. Gasparini, and E. Gasparini on December 14, 2017.

**Referred specimens.** Four specimens collected near Parque Estadual Paulo César Vinha, Setiba, municipality of Guarapari (20°36′23.01″S, 40°25′13.27″W; 3 m asl), Espírito Santo State, Brasil: CFBH 4112 (female) collected on March 16, 2002 by J.L. Gasparini and R.C. Bianchi; CFBH 9967 (female), CFBH 9968 (male), collected on April 18, 2004 by A.F. Béda, P. Landgref Filho, and M. Uetanabaro; CFBH 33358 (female) collected on April 3, 2005 by J.L. Gasparini, P.L.V. Peloso, and H. Moura.

**Diagnosis.** The new species is assigned to *Pseudopaludicola* by its phylogenetic position and by the presence of a tubercle on the forearm, anterolateral processes of the hyoid absent, posterolateral processes of hyoid reduced, and epicoracoid cartilages slightly superposed. The new species is characterized by: (1) SVL 11.7–14.6 mm in males, 14.0–16.7 mm in females; (2) body slender; (3) fingertips knobbed with a central groove; (4) hindlimbs short; (5) abdominal fold complete; (6) arytenoid cartilages wide; (7) prepollex with base and two segments; (8) prehallux with base and one segment; (9) frontoparietal fontanelle partially exposed; (10) advertisement call with one note, composed of two isolated pulses per call; (11) dominant frequency ranging from 4380–4884 Hz; 12) diploid chromosome number of 22; and 13) Ag-NORs on 8q subterminal.

**Comparison with other species.** *Pseudopaludicola restinga* sp. nov. can be distinguished from all other recognized species of *Pseudopaludicola* by a combination of external morphological, osteological, acoustic, chromosomal characters, and 16S rDNA sequences.

The smaller SVL of males (11.7–14.6; *n* = 21, Table 2) in *P. restinga* sp. nov. clearly separates it from *P. ameghini* (14.1–19.3, Pansonato et al., 2013), *P. giarettai* (16.2–18.0, Carvalho, 2012), and *P. ternetzi* (16.0–18.6, Lobo, 1996). In addition, the slender body of *P. restinga* sp. nov. differentiates this species from *P. ameghini* and *P. ternetzi* that have robust bodies (Lobo, 1995; Lobo, 1996; Pansonato et al., 2013), and also from *P. mineira* which has a more globular aspect (Lobo, 1994; Lobo, 1995).

The knobbed toe tips with central grooves separate *P. restinga* sp. nov. from the species of the *P. pusilla* group (*P. boliviana*, *P. ceratophyes*, *P. llanera*, *P. motorzinho*, and *P. pusilla*), which have T-shaped toe tips (Lynch, 1989; Lobo, 1995; Cardozo & Suárez, 2012; Pansonato et al., 2016).

The presence of a complete abdominal fold differentiates *P. restinga* sp. nov. from *P. falcipes*, in which the abdominal fold is incomplete or absent (Lobo, 1994; Lobo, 1995), while the absence of tubercles on the eyelids separates the new species from *P. hyleaustralis*, which has minute tubercles on the eyelids (Pansonato et al., 2012).

When the hind limb is stretched anteriorly, the tibio-tarsal articulation reaches the anterior border of the eye in *P. restinga* sp. nov., whereas in *P. jaredi*, *P. murundu*, and *P. saltica* the tibio-tarsal articulation extends past the tip of the snout (Lobo, 1994; Lobo, 1995; Toledo, 2010; Andrade et al., 2016).

The vocal sac in males, with dispersed or aggregated small spots (see variation), separates *P. restinga* sp. nov. from *P. atragula* (areolate vocal sac with dark reticulation; Pansonato et al., 2014a). In addition, the developed vocal sac with a central fold (Fig. 2B) separates *P. restinga* sp. nov. from *P. ibisoroca*, which has a poorly developed vocal sac without folds (Pansonato et al., 2016).

The wide larynx, with arytenoid cartilages encompassing almost the entire region between the posteromedial processes of the hyoid (similar to *P. mystacalis*), separates *P. restinga* sp. nov. from *P. ameghini*, *P. canga*, *P. ceratophyes*, *P. falcipes*, *P. giarettai*, *P. llanera*, *P. mineira*, *P. motorzinho*, *P. murundu*, *P. parnaiba*, *P. pocoto*, *P. pusilla*, *P. saltica*, and *P. ternetzi*, in which the larynx is composed of two small oblong shell-shaped arytenoid cartilages that encompass less than half of the space between the posteromedial processes of the hyoid (for comparison see Fig. 1B, Lobo, 1995; Fig. 3C, Roberto, Cardozo & Ávila, 2013).

The advertisement call, composed of pulsed notes, separates *P. restinga* sp. nov. from all species with non-pulsed notes: *P. canga* (Giaretta & Kokubum, 2003), *P. facureae* (Andrade & Carvalho, 2013), *P. giarettai* (Carvalho, 2012), *P. hyleaustralis* (Pansonato et al., 2012), and *P. parnaiba* (Roberto, Cardozo & Ávila, 2013)). The call with isolated pulses, separates *P. restinga* sp. nov. from *P. atragula*, *P. boliviana*, *P. ibisoroca*, *P. motorzinho* and *P. mystacalis*, which have advertisement calls with concatenated pulses (Márquez, De la Riva & Bosch, 1995; Duré et al., 2004; Pansonato et al., 2013; Pansonato et al., 2014a; Pansonato et al., 2014b; Pansonato et al., 2016). In addition, *P. restinga* sp. nov. differs from the other species with isolated pulsed notes by having a lower number of pulses per note (two pulses per note): *P. ameghini* (three to six pulses per note; Pansonato et al., 2013), *P. atragula* (nine to 36 pulses per note; Pansonato et al., 2013); *P. pocoto* (three pulses per note; Magalhães et al., 2014); and *P. ternetzi* (three to six pulses per note; Cardozo & Toledo, 2013). Likewise, the variable advertisement calls of *P. murundu* (two to six pulses per note; Toledo et al., 2010), *P. jaredi* (two to seven pulses per note; Andrade et al., 2016), and *P. saltica* (one to four pulses per note; Pansonato et al., 2013) contrast with *P. restinga* sp. nov., in which the call is always composed of two pulses per note.

A karyotype with 2*n* = 22 chromosomes separates *P. restinga* sp. nov. from: *P. ameghini*, *P. boliviana*, *P. ternetzi* (2*n* = 20); *P. atragula*, *P. canga, P. facureae*, *P. ibisoroca* (2*n* = 18); and *P. mystacalis* (2*n* = 16) (Cardozo et al., 2016 and citations therein).

From the other species with 2*n* = 22 chromosomes, the Ag-NORs on 8q subterminal separates *P. restinga* sp. nov. from *P. falcipes* (8q pericentromeric; Fávero et al., 2011; Cardozo et al., 2016); *P. mineira* (4p pericentromeric; Duarte et al., 2010); and *P. motorzinho* (8q intersticial; Fávero et al., 2011), while the absence of sex chromosomes in *P. restinga* sp. nov. distinguishes it from *P. saltica* (XX/XY chromosome sex determination; Duarte et al., 2010). The new species shares the 2*n* = 22 diploid number and the location of NORs with *P. jaredi*, *P. murundu*, and *P. saltica*. However, Pair 8 in *P. jaredi, P. murundu*, and *P. saltica* are telocentric, FN = 42 (Duarte et al., 2010; Andrade et al., 2016), while in *P. restinga* sp. nov. the Pair 8 is subtelocentric; FN = 44.

**Description of holotype.** Head longer than wide, CI = 1.14. Snout acuminate in dorsal view, protruding beyond the jaw in lateral view. Nostrils nearer to the tip of the snout than to the eyes. Internarial distance slightly less than interorbital distance (Table 2). Vocal sac developed and with a central fold. Tympanum not visible. Undifferentiated canthus rostralis, loreal region flat. Tongue entire, oval, posteriorly free, base unpigmented. Premaxillary and maxillary teeth present. Body slender, skin smooth, with scarce small and flattened glandular warts on the flanks. An X-shaped glandular fold is present in the interscapular region. Ventral region whitish, with small groups of dark spots on the throat, abdomen, and thigh. Abdominal fold complete. Antebrachial tubercle evident at the lateral margin of the forearms. Inner and outer metacarpal tubercles elongated and rounded, respectively. Nuptial pad light brown, covering the inner region of the inner metacarpal tubercle. Length of fingers III < II < IV < V; fingers with developed distal subarticular tubercles; fingertips knobbed and with a central groove. Arm speckled dorsally. Hindlimb with striped dorsal pattern and two dark bands. Inner surface of thighs with light brown background color finely spotted with black. Inner metatarsal tubercle well developed, curved distally, outer metatarsal tubercle conical and perpendicular to the plane of the foot. Tarsal fold curved, with a thickening at the middle of the tarsus simulating a tubercle. Toes marginally fringed. Length of toes IV > III > V > II > I; toes with tips not expanded laterally; toe tips knobbed with central grooves.

**Measurements of holotype (in millimeters).** SVL 14.9; HL 4.8; HW 4.2; ED 1.7; IOD 1.3; IND 1.2; TL 7.3; THL 7.1; END 1.1; NSD 0.7; FL 7.9; LII 1.1; LIII 1.4.

**Variation.** The type series and the rest of the specimens identified herein as *P. restinga* sp. nov. exhibit little variation (Fig. 3). However, almost all specimens from Restinga de Praia das Neves possess brownish dorsolateral bands on a dark brown dorsal background, brownish thighs with dark brown crossed bands, a densely spotted belly, and an urostylar vertebral line (CFBH 39231–2) that sometimes reaches the tip of the snout (CFBH 39228). All other specimens, the urostylar vertebral line is absent and the skin of the dorsum varies from smooth to somewhat warty, with dispersed small glands (Figs. 3A–3B, 3D).

The ventral region is usually whitish with dispersed groups of tiny dark spots (in about 60% of the specimens), or dense spotted areas covering the entire venter except for the posterior region of the abdomen (Fig. 3C), a condition present in 20% of the specimens. Males have light brown nuptial pads covering most of the inner metacarpal tubercle and exceeding the size of the subarticular tubercle of Finger II (Fig. 2A).

**Osteology.** Skull wider than long (Figs. 4A–4B). Maxillary arch complete. Alary processes of premaxillae directed dorsally, parallel to each other (frontal view). Premaxillae bearing 11 teeth and maxillae bearing 28 curved pedicellate teeth. Nasals narrow, ovoid, with irregular inner margins and ending in a posteriorly directed point. Nasals separated from each other and not overlapping the maxilla. In dorsal view, the nasals do not overlap the sphenethmoid. Anterior margin of the sphenethmoid not reaching the middle of the choanae; posterior margin W-shaped. In dorsal view, sphenethmoid short with a thin internasal septum. Frontoparietals with irregular inner margins, leaving the frontoparietal fontanelle partially visible. Exoccipitals separated by a wide mineralized band. Optic foramen large, ovoid, and located at the junction between the prootics and the sphenethmoid (exit of cranial nerves II, IV, and VI). Oculomotor foramen narrow in diameter, located posterior to the optic foramen (cranial nerve III). Prootic foramen oval, located posterior to the optic capsule (cranial nerve V). Jugular foramen with two holes (exit of cranial nerves IX and X). Pterygoids triradiate. Posterior rami of pterygoids longer than medial rami and separated from their respective quadrates; medial rami detached from the alae of the parasphenoid and supported by cartilage in the otic region of the skull. Anterior rami separated from the palatines. Quadratojugals reduced with evident anterior processes. Parasphenoid triradiate with the tip of the cultiform process being irregular. Parasphenoid alae perpendicular to the cultriform process. Neopalatines thin, overlapping the *pars fascialis* of the maxillae. Prevomers small, irregularly-shaped and without teeth. Squamosals with ventral rami wider at their ventral ends; zygomatic rami shorter than otic rami and with well-developed descending processes. Tympanic annuli cartilaginous, opened dorsally. Plectrum of uniform width. Operculum cartilaginous, rounded. Mandible with thin dentaries covering the lateral margins of Meckel’s cartilages. Inner part of Meckel’s cartilages covered by respective angular bones. Articular regions of the lower jaw cartilaginous, dentaries plain, without denticle-like structures.

Hyoid plate cartilaginous and with a mineralized median strip (Fig. 5A). Anteromedial processes of the hyoid short and divergent. Anterolateral process absent. Posterolateral processes barely developed. Posteromedial processes with cartilaginous ends united to the cricoid cartilage by ligaments. The larynx encompasses almost the entire region between the posteromedial processes of the hyoid.

Arytenoid cartilages of the larynx develop as two oblong shells. The cricoid cartilage forms a complete ring (Figs. 5B–5C) with short and thin bronchial process; undeveloped esophageal process; well-developed muscular processes; and poorly developed cardiac and articular processes.

Vertebral column with eight procoelous, non-imbricate presacral vertebrae (Fig. 6A). Cotylar facets of the atlas separated (type I of Lynch, 1971). Atlas body widest among the vertebrae. In dorsal view, the anterior margin of the atlas contacts Presacral II through a wide neural process. Anterior portion of the illium articulates with the ventral surface of the sacral diapophyses. Pubis cartilaginous. Sacral diapophyses narrow and not expanded. Urostyle with a well-developed dorsal spine, exit of spinal nerve X not visible. Urostylar articulation bicondylar.

Pectoral girdle arciferal (Fig. 6B), with the left epicoracoid slightly superimposed on the right, in ventral view. Clavicle thin, posterior margin concave, separated from the acromial region of the scapula by an anterior projection of the procoracoid. Scapula with poorly developed pars acromialis and pars glenoidalis. Anterior process of the suprascapula present. Omosternum mineralized, with slightly expanded terminal end. Sternum simple, not bifurcated, with osseous mesosternum and irregularly expanded cartilaginous xiphisternum.

Humerus with evident deltoid crest. Radius-ulna fused, leaving a narrow groove between them beginning at the union of the carpal elements and extending for one third of the length of both bones. Carpus composed of five elements: radiale, ulnare, Y-element, Distal Carpal V–IV–III, and Distal Carpal II. Metacarpals III, IV, and V, articulating with Distal Carpal V–IV–III; Metacarpal II with Distal Carpal II; base of prepollex articulating with the Y-element and Distal Carpal II. Prepollex with one osseous base and two mineralized segments. Phalangeal formula: 2–2–3–3. Toe tips knobbed with central groove (Fig. 6C).

Hindlimbs with the tibia-fibula fused at their distal ends. Tarsus composed of centrale, Distal Tarsal I, and Distal Tarsal II–III. Distal Tarsal I is the smallest and articulates with the other two elements. The centrale articulates with the prehallux base and Metatarsal I. Distal Tarsal II–III articulating with Metatarsals II and III, in contact with Metatarsal IV. Metatarsals IV and V articulate directly with the astragalus. The prehallux has one osseous base and one cartilaginous segment. Phalangeal formula: 2-2-3-4-3. Toe tips knobbed with central groove (Fig. 7D).

In the specimen examined, there are some osseous lateral projections at the anterior extremities of the metacarpal epiphysis and some phalanges. In the forelimb, in dorsal view, there is a small rounded sesamoid bone over Distal Carpal V–IV–III, embedded in the tendon of the muscle carpi ulnaris. In ventral view, the palmar sesamoid, embedded in the m. digitorum longus, has an irregular quadrangular shape. Glide sesamoids are also present in the flexor tendons at the junction of the proximal and medial phalanges of all fingers. Hind limb with osseous lateral projections at the epiphysis of some metatarsals and phalanges. Sesamoid graciella (at the joint between femur-tibia fibula, embedded at the tendon of the m. gracilis major), sesamoid cartilage (in the tendon of the m. plantaris profundus) and two ovoid plantar sesamoids of similar size are in contact with the tendons that form the aponeurosis plantaris. Additionally, near the distal end of the sacral diapophyses there are a couple of elongated sesamoid bones (sesamoid of the sacral vertebrae) of similar width, partially overlapping the diapophyses.

**Advertisement call.** Males of *Pseudopaludicola restinga* sp. nov. call during both day and night from along the margins of shallow temporary ponds, usually after heavy rainfall. The advertisement call (Fig. 7) consists of a repeated sequence of single notes, which are composed of two isolated pulses without frequency modulation. The length of each call is of 47 ms (39–58 ms), emitted at intervals of 83.26 ms (64–133 ms). Mean duration of the first pulse is 4.18 ms (3–5 ms) and of the second 5.27 ms (4–7 ms); mean interpulse interval is 38.62 ms (33–50 ms); mean rate of emission is 21.3 calls/s (17.2–25.6); and mean dominant frequency is 4592.32 Hz (4380–4884 Hz).

**Cytogenetic analysis.** *Pseudopaludicola restinga* sp. nov. has a diploid number of 22 chromosomes (2*n* = 2*x* = 22; FN = 44). The pairs 1–3, 5, 7, and 9–11 are metacentric; pairs 4 and 6 submetacentric, while pair 8 is subtelocentric (Fig. 8, Table 3). The C-banding pattern is distributed on all the centromeres of the chromosome complement, at the subterminal region of the long arm pair 8 and 9 (Fig. 9B). The NORs are located on 8q subterminal, in coincidence with the usually evident secondary constrictions; in all examined specimens no heteromorphic sex chromosomes were identified with the staining techniques used.

**Table 3:** Morphometric analysis of chromosomes of *Pseudopaludicola restinga* sp. nov.

Species												
		**1**	**2**	**3**	**4**	**5**	**6**	**7**	**8**	**9**	**10**	**11**
***Pseudopaludicola restinga*sp. nov.**	RL	15.0	14.3	10.2	11.0	10.1	9.5	8.7	7.5	5.2	4.8	3.4
	AR±SD	1.33 ± 0.14	1.02 ± 0.01	1.65 ± 0.16	1.76 ± 0.16	1.59 ± 0.12	2.23 ± 0.28	1.30 ± 0.16	4.06 ± 0.65	1.19 ± 0.03	1.33 ± 0.21	2.05 ± 0.32
	CT	**m**	**m**	**m**	**Sm**	**m**	**Sm**	**m**	**St**	**m**	**m**	**Sm**

**Notes.**

RL relative length AR arm ratio CT chromosomic type m metacentric sm submetacentric st subtelocentric

**Etymology.** The word “restinga” has an unclear origin in Brazilian Portuguese, but it is the proper name of the vegetation near the sea where the new species occurs: the restingas. Apparently, “res” comes from Latin, meaning “thing” and “tinga” comes from the Tupi indigenous language, meaning “white”, probably an allusion to the clear sandy soil of this formation. Here, restinga is used as a noun in apposition.

**Distribution.** *Pseudopaludicola restinga* sp. nov. is known from six municipalities in Espírito Santo State, Brazil (Fig. 9): Serra (type locality), Guarapari, Presidente Kennedy, Vitória (Reserva Ecológica Municipal Mata Paludosa), Vila Velha (Morada Interlagos and Vale Encantado lagoon), and Itapemirim (Lagoa das Sete Pontas or Lagoa Guanandy).

**Natural history notes and conservation insights.** This species can be found between dunes or at the borders of lagoons in wet or flooded places, mostly after rainfall events. The individuals were found vocalizing on the mud during the day. In addition, in the rainy season males also call in the evening and night. The restingas belong to the Atlantic Forest biome, a global biodiversity “hotspot” (Myers et al., 2000). In the restingas, the plants form paludous or herbaceous forests, but the constant disturbance of this habitat, induced by man, has led to the loss of most of their original area (Rocha et al., 2003). In concordance with the economic expansion of Brazil from 2000 to 2014, multiple infrastructure projects were developed on coastal areas of Espírito Santo, particularly port activity, and oil and gas exploitation. As a consequence, multiple pristine areas have been severely affected, including Restinga de Praia das Neves. The presence of a new anuran species in the restingas along the coastal areas of Espírito Santo contributes to the knowledge of the richness of vertebrate biodiversity of this fragile ecosystem, which requires conservation policies capable of maintaining and preserving the biological heritage of the region and of the country.

## Conclusions

Despite increasing taxonomic knowledge regarding *Pseudopaludicola*, multiple populations are still of uncertain taxonomic status. In this sense, the specimens assigned to *P.* aff. *canga* (Duarte et al., 2010), from Barreirinhas, Maranhão, were assigned to *P. canga* by Pansonato et al. (2012). However, the same specimens were named as *P.* sp. 3 (aff. *canga*) in Veiga-Menoncello et al. (2014), and as *P*. aff. *canga* from Barreirinhas in Pansonato et al. (2016), which indicates the need for reevaluation of the taxonomic status of this population. Another taxonomic question involves the validity of *P. parnaiba*, a species that was questioned by Carvalho et al. (2015) based on acoustic comparisons. Yet another taxonomic problem is the assignment of specimens to *P. ternetzi* by Cardozo et al. (2016), which were also almost simultaneously assigned to *P. ameghini* by Lavilla et al. (2016) using external morphology.

The data used to discriminate *Pseudopaludicola* species are variable, and the utility of such evidence should not be generalized. Advertisement calls were proposed as an important tool for discriminating species of *Pseudopaludicola* (Pansonato et al., 2014b). However, species described and supported by advertisement call, such as *P. murundu* and *P. serrana*, were later considered synonyms by molecular data and the reanalysis of the advertisement calls (Pansonato et al., 2014b; Veiga-Menoncello et al., 2014). In addition, *P. jaredi*, *P. saltica*, and *P. murundu* share overlapping call parameters (Andrade et al., 2016). Likewise, overlapping acoustic parameters among *P. falcipes*, *P. mineira*, and *P. restinga* sp. nov. highlight the importance of considering other sources of evidence for the discrimination of species, since these three taxa are monophyletic and diagnosable by characters of external morphology, osteology, cytogenetics, and DNA sequences (see diagnosis). Moreover, although rDNA sequences generally present high interspecific differences in *Pseudopaludicola*, some sister species pairs show significantly lower genetic differences: *P. ameghini* and *P. ternetzi* have 16S divergence of 1.6%, and *P. saltica* and *P. murundu* 2.5% (Pansonato et al., 2014a). Conversely, *P. mystacalis*, throughout its broad distribution, reaches differences somewhat higher than 3%. It is clear that genetic divergence could vary among clades, as was previously shown for other anuran groups (i.e., Lötters et al., 2009, Blotto et al., 2013; Pereyra et al., 2016), so observed genetic divergences should be taken with caution. In *P. restinga* sp. nov., the genetic divergence of 4.41% with regard to *P. pocoto* (the most closely-related taxon, Fig. 1), and the presence of a wide larynx, in combination with other morphological and acoustic characters (see diagnosis), support the discrimination of this new taxon as an independent lineage.

##  Supplemental Information

10.7717/peerj.4766/supp-1 Supplemental Information 1 16S partial sequence provided in this study

## Additional Information and Declarations

### DNA Deposition

The following information was supplied regarding the deposition of DNA sequences: The 16S partial sequence described here are accesible via GenBank accession numbers MG825764, MG825766, MG825767.

### Data Availability

The following information was supplied regarding data availability: The research in this article did not generate any data or code. All new data is present in the manuscript and the ribosomal sequences used are present in Genbank.

### New Species Registration

The following information was supplied regarding the registration of a newly described species: *Pseudopaludicola restinga* sp. nov. urn:lsid:zoobank.org:act:776C919B-CD34-49AD-8413-FC480407B859 Publication lsid urn:lsid:zoobank.org:pub:114E0628-F00E-42B3-A98E-CB157DCA1CF1.
