# Supplementary material for: A new species of Pseudopaludicola (Anura, Leiuperinae) from Espírito Santo, Brazil
Source: PeerJ. 2018 May 16;6:e4766. doi: 10.7717/peerj.4766 (PMC5960265; doi:10.7717/peerj.4766)
Supplement: Supplemental Information 1 [file peerj-06-4766-s001.docx]

**#Accession** Sequence ID Release Date

**MG825764** CFBHT_6678_16S Feb 22, 2019

>CFBHT_6678_16S *Pseudopaludicola sp nova* <restinga>

GCTAATTATCTAGCCCCAACACTACACATGAACACCACCTTCCTCACACATCTTCCCTAAATCATTTTCATAATTTAGTAGAGGGGATCAAAAAATTATTTAGGAGCTATAAAAATAGTACCGCAAGGGAAAGATGAAATATTAATGAAATATTCTCAAGCAAATAAAAGTAGAGACTAAGCCTCGTACCTTTTGCATCATGGTTTAACAAGTTCAATCAAGCAAAAAGATTTTCAGTTTGACCTCCCGAAACTAAGCGAGCTACTTAAAGACAGCTTTTAGAGCAAACCCATCTCTGTAGCAAAAGAGTGGAAAGATCATTAAGTAGAGGTGACAAACCTACCGAGCATAGTGATAGCTGGTTGCTTAAGAAAAGGATCTCAGTTCAACCTAAATCTACTTTTACAAGCCCAAGTACATAATTTAGAGCAATTCTAACAAGGTACAGCCTGTTAGAACCAGGATACAACCTTAACTAATGGGTAAACACATCTCCCCTGCAACAAAGTGGGCCTAAAAGCAGCCATCTTTAAAACAGCGTCAAAGCTTAATTGCAGTATAGATAAATCTCACACCCTAAATTCAACCCTTTATTAGTATTAAGCAATTCTATACAATTATAGAAATTTTTATGTTAAAACTAGTAACAAGAATAAGATATTCTCTAGAATGCAAGTGTAAGCCAGCCTAGGACAACACACTGACAATTAACATTTTTAACACCTTAGTAGTAACATATCAAGAAAACCCTACTCACTCATATGTTAATCTAACACCAGAGCATTTCAAGAAAGATTAAAAGAAAAAGAAGGAACTCGGCAAACATTAACCTCGCCTGTTTACCAAAAACATCGCCTCTTGCACAACCATAAGAGGTCCAGCCTGCCCAGTGACTCTGTTCAACGGCCGCGGTATCCTAACCGTGCAAAGGTAGCGCAATCACTTGTTCTTTAAATAAGGACTAGTATGAATGGCACCACGAAGGTTATACTGTCTCCTCTTTCTAATCAGTGAAACTAATCTTCCCGTGAAGAAGCGGGAATAACCATATAAGACGAGAAGACCCTATGGAGCTTTAAACTAAGCAACAAATATCTCCCCACTTAAAAGACTTAAGAGTAATTAAAATTAACCAGATATTATGATTTCTAGTTTTAGGTTGGGGTGACCATGGAGTACAATTTAACCTCCATGCCGAATGATTATAAAAATCTAAGCCACGAACAACAATTCCAAGCATCAACACTTTGACGTCTATTGACCCAATTTTTGATCAACGAACCAAGTTACCCTAGGGATAACAGCGCAATCCACTTCAAGAGTTCATATCGACAAGTGGGTTTACGACCTCGATGTTGGATCAGGGTATCCTAGTGGTGCAGCCGCTACTAAAGGTTCGTTTGTACAACGATTAAAACCCT

**#Accession** Sequence ID Release Date

**MG825766** CFBHT_6679_16S Feb 22, 2019

>CFBHT_6679_16S *Pseudopaludicola* sp nova <restinga>

GCTAATTATCTAGCCCCAACACTACACATGAACACCACCTTCCTCACACATCTTCCCTAAATCATTTTCATAATTTAGTAGAGGGGATCAAAAAATTATTTAGGAGCTATAAAAATAGTACCGCAAGGGAAAGATGAAATATTAATGAAATATTCTCAAGCAAATAAAAGTAGAGACTAAGCCTCGTACCTTTTGCATCATGGTTTAACAAGTTCAATCAAGCAAAAAGATTTTCAGTTTGACCTCCCGAAACTAAGCGAGCTACTTAAAGACAGCTTTTAGAGCAAACCCATCTCTGTAGCAAAAGAGTGGAAAGATCATTAAGTAGAGGTGACAAACCTACCGAGCATAGTGATAGCTGGTTGCTTAAGAAAAGGATCTCAGTTCAACCTAAATCTACTTTTACAAGCCCAAGTACATAATTTAGAGCAATTCTAACAAGGTACAGCCTGTTAGAACCAGGATACAACCTTAACTAATGGGTAAACACATCTCCCCTGCAACAAAGTGGGCCTAAAAGCAGCCATCTTTAAAACAGCGTCAAAGCTTAATTGCAGTATAGATAAATCTCACACCCTAAATTCAACCCTTTATTAGTATTAAGCAATTCTATACAATTATAGAAATTTTTATGTTAAAACTAGTAACAAGAATAAGATATTCTCTAGAATGCAAGTGTAAGCCAGCCTAGGACAACACACTGACAATTAACATTTTTAACACCTTAGTAGTAACATATCAAGAAAACCCTACTCACTCATATGTTAATCTAACACCAGAGCATTTCAAGAAAGATTAAAAGAAAAAGAAGGAACTCGGCAAACATTAACCTCGCCTGTTTACCAAAAACATCGCCTCTTGCACAACCATAAGAGGTCCAGCCTGCCCAGTGACTCTGTTCAACGGCCGCGGTATCCTAACCGTGCGAAGGTAGCGCAATCACTTGTTCTTTAAATAAGGACTAGTATGAATGGCACCACGAAGGTTATACTGTCTCCTCTTTCTAATCAGTGAAACTAATCTTCCCGTGAAGAAGCGGGAATAACCATATAAGACGAGAAGACCCTATGGAGCTTTAAACTAAGCAACAAATATCTCCCCACTTAAAAGACTTAAGAGTAATTAAAATTAACCAGATATTATGATTTCTAGTTTTAGGTTGGGGTGACCATGGAGTACAATTTAACCTCCATGCCGAATGATTATAAAAATCTAAGCCACGAACAACAATTCCAAGCATCAACACTTTGACGTCTATTGACCCAATTTTTGATCAACGAACCAAGTTACCCTAGGGATAACAGCGCAATCCACTTCAAGAGTTCATATCGACAAGTGGGTTTACGACCTCGATGTTGGATCAGGGTATCCTAGTGGTGCAGCCGCTACTAAAGGTTCGTTTGTTCAACGATTAAAACCCT

**#Accession** Sequence ID Release Date

**MG825767** CFBHT_19566_16S Feb 22, 2019

>CFBHT_19566_16S Pseudopaludicola sp nov

GCTAATTATCTAGCCCCAACACTACACATGAACACCACCTTCCTCACACATCTTCCCTAAATCATTTTCATAATTTAGTAGAGGGGATCAAAAAATTATTTAGGAGCTATAAAAATAGTACCGCAAGGGAAAGATGAAATATTAATGAAATATTCTCAAGCAAATAAAAGTAGAGACTAAGCCTCGTACCTTTTGCATCATGGTTTAACAAGTTCAATCAAGCAAAAAGATTTTCAGTTTGACCTCCCGAAACTAAGCGAGCTACTTAAAGACAGCTTTTAGAGCAAACCCATCTCTGTAGCAAAAGAGTGGAAAGATCATTAAGTAGAGGTGACAAACCTACCGAGCATAGTGATAGCTGGTTGCTTAAGAAAAGGATCTCAGTTCAACCTAAATCTACTTTTACAAGCCCAAGTACATAATTTAGAGCAGTTCTAACATGGTACAGCCTGTTAGAACCAGGATACAACCTTAACTAATGGGTAAACACATCTCCCCTGCAACAAAGTGGGCCTAAAAGCAGCCATCTTTAAAACAGCGTCAAAGCTTAATTGCAGTATAGATAAATCTCACACCCTAAATTCAACCCTTTATTAGTATTAAGCAATTCTATACAATTATAGAAATTTTTATGTTAAAACTAGTAACAAGAATAAGATATTCTCTAGAATGCAAGTGTAAGCCAGCCTAGGACAACACACTGACAATTAACATTTTTAACACCTTAGTAGTAACATATCAAGAAAACCCTACTCACTCATATGTTAATCTAACACCAGAGCATTTCAAGAAAGATTAAAAGAAAAAGAAGGAACTCGGCAAACATTAACCTCGCCTGTTTACCAAAAACATCGCCTCTTGCACAACCATAAGAGGTCCAGCCTGCCCAGTGACTCTGTTCAACGGCCGCGGTATCCTAACCGTGCgAAGGTAGCGCAATCACTTGTTCTTTAAATAAGGACTAGTATGAATGGCACCACGAAGGTTATACTGTCTCCTCTTTCTAATCAGTGAAACTAATCTTCCCGTGAAGAAGCGGGAATAACCATATAAGACGAGAAGACCCTATGGAGCTTTAAACTAAGCAACAAATATCTCCCCACTTAAAAGACTTAAGAGTAATTAAAATTAACCAGATATTATGATTTCTAGTTTTAGGTTGGGGTGACCATGGAGTACAATTTAACCTCCATGCCGAATGATTATAAAAATCTAAGCCACGAACAACAATTCCAAGCATCAACACTTTGACGTCTATTGACCCAATTTTTGATCAACGAACCAAGTTACCCTAGGGATAACAGCGCAATCCACTTCAAGAGTTCATATCGACAAGTGGGTTTACGACCTCGATGTTGGATCAGGGTATCCTAGTGGTGCAGCCGCTACTAAAGGTTCGTTTGTtCAACGATTAAAACCCT
